# Divergent coronary flow responses to uridine adenosine tetraphosphate in atherosclerotic ApoE knockout mice

**DOI:** 10.1007/s11302-017-9586-z

**Published:** 2017-09-20

**Authors:** Bunyen Teng, Hicham Labazi, Changyan Sun, Yan Yang, Xiaorong Zeng, S. Jamal Mustafa, Zhichao Zhou

**Affiliations:** 1 Department of Physiology and Pharmacology, Clinical and Translational Science Institute, West Virginia University, Morgantown, WV USA; 2 Present Address: Coagulation and Blood Research Task Area, US Army Institute of Surgical Research, San Antonio, TX USA; 3 Present Address: Center for Cardiovascular Research and The Heart Center, The Research Institute at Nationwide Children’s Hospital, Columbus, OH USA; 4 Present Address: Molecular Vascular Medicine, Department of Medicine, Karolinska University Hospital, Karolinska Institutet, Stockholm, Sweden; 5 Key Laboratory of Medical Electrophysiology of Ministry of Education, Collaborative Innovation Center for Prevention and Treatment of Cardiovascular Disease, Institute of Cardiovascular Research, Southwest Medical University, Luzhou, China; 6 Division of Cardiology, Department of Medicine, Karolinska University Hospital, Karolinska Institutet, 17176 Stockholm, Sweden

**Keywords:** Up_4_A, Atherosclerosis, Coronary flow, P2X_1_R, Vasoconstriction

## Abstract

Uridine adenosine tetraphosphate (Up_4_A) exerts potent relaxation in porcine coronary arteries that is reduced following myocardial infarction, suggesting a crucial role for Up_4_A in the regulation of coronary flow (CF) in cardiovascular disorders. We evaluated the vasoactive effects of Up_4_A on CF in atherosclerosis using ApoE knockout (KO) mice ex vivo and in vivo. Functional studies were conducted in isolated mouse hearts using the Langendorff technique. Immunofluorescence was performed to assess purinergic P2X_1_ receptor (P2X_1_R) expression in isolated mouse coronary arteries. In vivo effects of Up_4_A on coronary blood flow (CBF) were assessed using ultrasound. Infusion of Up_4_A (10^−9^–10^−5^ M) into isolated mouse hearts resulted in a concentration-dependent reduction in CF in WT and ApoE KO mice to a similar extent; this effect was exacerbated in ApoE KO mice fed a high-fat diet (HFD). The P2X_1_R antagonist MRS2159 restored Up_4_A-mediated decreases in CF more so in ApoE KO + HFD than ApoE KO mice. The smooth muscle to endothelial cell ratio of coronary P2X_1_R expression was greater in ApoE KO + HFD than ApoE KO or WT mice, suggesting a net vasoconstrictor potential of P2X_1_R in ApoE KO + HFD mice. In contrast, Up_4_A (1.6 mg/kg) increased CBF to a similar extent among the three groups. In conclusion, Up_4_A decreases CF more in ApoE KO + HFD mice, likely through a net upregulation of vasoconstrictor P2X_1_R. In contrast, Up_4_A increases CBF in vivo regardless of the atherosclerotic model.

## Introduction

Ischemic heart disease is characterized by the development of coronary atherosclerosis and is one of the leading causes of death worldwide [1, 2]. The danger of atherosclerosis is thromboembolism, which evolves from atherosclerotic plaques or thrombotic vessel occlusion and can lead to cardiac ischemia and infarction [2]. Subsequent tissue damage increases the release of extracellular nucleotides and nucleosides, which activate and alter purinergic signaling [3]. Pharmacological interventions using ATP, adenosine, and, more commonly, the purinergic P2Y_12_ receptor antagonist clopidogrel, have been used for treating patients with coronary artery disease [4].

Uridine adenosine tetraphosphate (Up_4_A) was identified as a novel vasoactive factor endogenously released from the endothelium [5, 6]. Up_4_A is the first dinucleotide found in living organisms that contains both purine and pyrimidine moieties, allowing Up_4_A to exert its vascular influence through both the purinergic P1 and P2 receptors [6, 7]. Several in vitro studies showed that Up_4_A induced vascular contraction in rat renal arteries and mouse aortas through the P2X_1_ receptor (P2X_1_R) [5, 8, 9], in rat aortas through the P1 receptor (P1R) and P2X receptor (P2XR) [10], and in rat pulmonary arteries through the P2Y receptor (P2YR) [11]. Vasoconstriction was also observed in renal arterioles [12] and rat mesenteric arteries [13]. In contrast to vasoconstrictor influence, Up_4_A produced vascular relaxation in rat aortas [10, 14], rat kidney [15] and porcine coronary microvessels when the tone is elevated [6]. Interestingly, the vascular effects and potency vary from mice to rats within the same type of arterial segments. Thus, Up_4_A produces a more pronounced contraction in rat aortas compared to mouse aortas at basal tone [9, 10], whereas Up_4_A induces a concentration-dependent relaxation in rat aortas but a potent contraction in mouse aortas when the vessel tone is elevated [9, 10]. These findings suggest that the vascular effects of Up_4_A may not only depend on different vascular beds but also on the species studied.

Observations that intra-aortic injection of Up_4_A increases mean arterial blood pressure in intact animals [5], plasma concentrations of Up_4_A in juvenile hypertensive subjects were significantly elevated [16], and that the coronary vasodilator response to Up_4_A was blunted in swine following myocardial infarction [17] suggest a role for Up_4_A in the pathogenesis of cardiovascular diseases, including the regulation of coronary vasomotor tone. However, the coronary vascular response to Up_4_A in atherosclerosis has not been explored.

The present study aimed to determine whether the effect of Up_4_A on coronary flow (CF) is altered in atherosclerosis and to evaluate the possible role of purinergic receptor subtypes using Langendorff-perfused isolated hearts from the ApoE knockout (KO) mice [18]. Since the effect of Up_4_A on coronary blood flow (CBF) in vivo is still lacking, we also aimed to investigate the effect of Up_4_A on CBF in vivo in atherosclerotic mice using Doppler echocardiography.

## Methods

All experimental protocols were performed according to the West Virginia University guidelines and with the approval of the Animal Care and Use Committee.

### Animals

ApoE KO mice with a C57BL/6 background were purchased from The Jackson Laboratory (Bar Harbor, ME, USA) and bred in our animal facility. At 8 weeks of age, one group of ApoE KO mice were fed a high-fat diet (HFD) (0.2% cholesterol, 21.2% fat, Harlan Teklad, TD88137) for 12 weeks to accelerate atherosclerotic lesion formation. Another group of ApoE KO mice and wild-type (WT) mice (C57BL/6 background) were maintained on a standard laboratory diet. All animals were kept in cages with 12:12 h light/dark cycles with access to water ad libitum. According to our previous findings [19–21], ApoE KO mice with HFD treatment for 12 weeks exhibited more extensive atherosclerotic lesions and greater total cholesterol levels than ApoE KO mice with the standard diet for the same period, the latter of which was significantly more extensive compared to WT mice. Atherosclerotic lesions were also formed in left coronary arteries of ApoE KO + HFD mice (unpublished).

### Langendorff-perfused mouse heart preparations

Mice (20–22 weeks) of either sex were anesthetized with pentobarbital sodium (50 mg/kg, i.p.). Mice were weighed before hearts were rapidly removed into heparinized (5 U/mL) ice-cold Krebs-Henseleit buffer containing (in mM) 119 NaCl, 11 glucose, 22 NaHCO_3_, 4.7 KCl, 1.2 KH_2_PO_4_, 1.2 MgSO_4_, 2.5 CaCl_2_, 2 pyruvate, and 0.5 EDTA. After removal of the surrounding tissue, the aorta was rapidly cannulated with a 20-gauge, blunt-ended needle, and the heart was continuously perfused with 37 °C buffer bubbled with 95% O_2_/5% CO_2_ at a constant perfusion pressure of 80 mmHg [22, 23]. Subsequently, through an opening in the left atrium, a fluid-filled balloon made of plastic wrap was inserted into the left ventricle across the mitral valve. The balloon was connected to a pressure transducer for continuous measurement of left ventricular developed pressure (LVDP) and heart rate (HR). The heart was then immersed in a water-jacketed perfusate bath maintained at 37 °C. The viability of the hearts was checked by a 15-s occlusion of inflow (a twofold increase). CF was continuously measured with an ultrasonic flow probe (Transonic Systems, Ithaca, NY, USA) placed in the aortic perfusion line. A PowerLab Chart data acquisition system (AD Instruments, Colorado Springs, CO, USA) was used for data acquisition. Hearts were allowed to equilibrate for 30 min before starting experimental protocols.

### Langendorff experimental protocols

After equilibrium, baseline CF, HR, and LVDP were measured. Up_4_A concentration response curves (10^−9^−10^−5^ M) were acquired in perfused hearts from WT, ApoE KO, and ApoE KO + HFD mice. Each concentration of Up_4_A was infused for 5 min, followed by a minimum of 5 min of perfusion for washout. Since Up_4_A-induced CF changes in WT mice were comparable to ApoE KO mice, in separate experiments, the P2X_1_R antagonist MRS2159 (30 μM [24]; Sigma-Aldrich, St. Louis, MO, USA) was perfused for 15 min before acquiring Up_4_A concentration response curves (10^−8^−10^−5^ M) in perfused hearts only from ApoE KO and ApoE KO + HFD mice. MRS2159 was continuously infused throughout the entire experiment at a rate of 1% of the CF through an injection port directly proximal to the aortic cannula using a microinjection pump (Harvard Apparatus, Holliston, MA, USA). Although MRS2159 predominantly blocks P2X_1_R it was designed for, it cannot be excluded entirely that other purinergic receptors were, to some extent, affected. Careful evaluation of selectivity of MRS2159 is necessary to properly interpret the results in the present study. In addition to P2X_1_R, evidence showed that MRS2159 affected P2X_2_R, P2X_2/3_R, and P2Y_1_R in 1321N1 cells [25] and P2X_7_R in 1321N1 cells and erythrocytes [25, 26]. However, the information on specificity of MRS2159 at the intact tissue level is currently lacking.

### Fluorescence immunostaining for the P2X_1_R in isolated mouse coronary arteries

Left coronary arteries (70–150 μm) from mouse hearts of WT, ApoE KO, and ApoE KO + HFD were isolated and fixed with 2% ice-cold paraformaldehyde for 30 min, then permeabilized for 10 min with 0.1% Triton X-100. The vessels were then blocked with 5% goat serum for 1 h before overnight incubation at 4 °C with rabbit anti-P2X_1_R (1:300 dilution; Alomone Labs, Israel) antibodies. The specificity of the antibodies has been tested in our previous study where a band of 55 kDa was observed [9]. The vessels were washed for 1 h with PBS and incubated for 2 h with PBS buffer containing Alexa 488-conjugated goat anti-rabbit secondary antibodies and DRAQ5 (a nuclear stain, 1 μM; Invitrogen). The vessels were washed again for 1 h with PBS and mounted on slides for imaging. A Zeiss LSM 510 confocal microscope was used to collect images from two randomly selected vessel areas. Each stack of images was acquired by optical sectioning at successive *x*–*y* focal planes with a vertical depth of 1 μm using a Zeiss objective (40/1.30, oil DIC, EC Plan-Neofluar, Thormwood, NY, USA) and a 1024 × 1024 scanning format. The mean fluorescence intensity (FI) of each stack of regions of interest (ROIs) that covered the area of individual endothelial cells (ECs, determined by the shape of the nucleus that longitudinally oriented along the vessel axis) and smooth muscle cells (SMCs, cells with a nucleus perpendicularly oriented along the vessel axis, Fig. 3a) was quantified using ImageJ. After subtraction of background signal, a mean of the FI averaged from three ROIs of each vessel segment was calculated and was presented in arbitrary units (AU).

### In vivo assessment of echocardiography and CBF Doppler measurement

In vivo echocardiograph assessments in mice were performed in accordance with our previously published methods [27, 28]. Briefly, each mouse was anesthetized in an induction chamber with inhalant isoflurane at 3% in 100% oxygen. When fully anesthetized, the mouse was transferred to dorsal recumbency, placed on a heated imaging platform, and maintained at 1–1.25% isoflurane for the duration of the experiment. A rectal probe was used to monitor the temperature of the mouse. The hair of the mouse chest was carefully removed, and warm electrode gel was applied to the limb leads, allowing for an electrocardiogram and the respiration rate to be recorded during ultrasound imaging. Ultrasound images were acquired using an MS550D transducer (22–55 MHz) on the Vevo2100 Imaging System (Visual Sonics, Toronto, Canada). Placing the transducer to the left of the sternum allowed us to obtain images of the aortic outflow tract, apex of the heart, and left ventricle along its longest axis (i.e., long-axis B-mode images). Once all long-axis B-mode images were attained, the transducer was rotated 90 ° to acquire short-axis B-mode images at the mid-papillary muscle level. Afterward, the transducer was moved up until the left coronary artery was visible to measure the size of the vessel. Then, the transducer was rotated back to the long-axis parasternal view with the probe lateralized and the ultrasound beam anteriorly tilted. In this image window, the entire left coronary artery, from the aortic sinus to the distal branch site, could be visualized using color Doppler echocardiography. The course of the left coronary artery was typically parallel to the Doppler beam, which facilitated Doppler measurements without any angle correction. Subsequently, the system was switched to pulse-wave Doppler mode with a gate size of 0.065 mm. CBF signals were identified on the Doppler spectral display by flow toward the probe, peaking in early diastole and then decaying and being minimal during systole as illustrated in Fig. 4a. Bolus injection of Up_4_A (0.04 mg/mouse, approximately 1.6 mg/kg) was made through the femoral vein. Flow velocity measurements were made at the same vessel site at baseline and during Up_4_A-mediated CF. Measurements were averaged from three consecutive cardiac cycles.

CBF was calculated using this formula: Flow_CBF_(mL/min) = ((π/4) × *D*2 × VTI × HR)/1000 [29] where *D* is the internal coronary diameter (in mm) measured in B-mode ultrasound images, VTI is the velocity-time-integral (in mm), or area under the curve of the Doppler blood flow velocity tracing, and HR is heart rate. Cardiac function and coronary artery size were measured again after the maximal Up_4_A effect was achieved.

### Statistical analysis

Langendorff baseline data for WT, ApoE KO, and ApoE KO + HFD groups were compared using one-way ANOVA followed by post hoc analysis using Bonferroni’s test. The Up_4_A concentration response curves performed during the Langendorff technique among different groups of mice and the effects of drug treatment on concentration response curves of Up_4_A were analyzed using two-way ANOVA for repeated measures. Since the absolute CF changes proportionally with heart mass, CF was presented as milliliter/minute/gram wet heart weight [22]. With regard to imaging, the mean FI of each ROI (including those in both ECs and SMCs) was calculated by subtraction of the background signal, and changes in FI were presented in AU. Image data were analyzed using one-way ANOVA followed by post hoc analysis using Bonferroni’s test. Paired *t* tests were used to compare the Up_4_A effect on CBF in vivo in paired conditions. All the data are presented as mean ± SEM; *n* represents the number of animals unless otherwise indicated. Statistical significance was accepted when *P* < 0.05.

## Results

### Baseline functional data in isolated WT, ApoE KO, and ApoE KO + HFD mouse hearts

Table 1 summarizes the baseline data for CF, HR, and LVDP in WT, ApoE KO, and ApoE KO + HFD mice after 30 min of equilibration of isolated hearts. Compared to age-matched ApoE KO or WT groups, ApoE KO + HFD mice exhibited significant increases in BW and HW (*P* < 0.05). However, the heart to body weight ratio was not significantly different among the three groups. There were no significant differences in baseline CF, HR, and LVDP among the three groups (*P* > 0.05, by one-way ANOVA, Table 1).

**Table 1:** Basal characteristics for isolated perfused mouse hearts

	WT (*n* = 6)	ApoE KO (*n* = 9)	ApoE KO + HFD (*n* = 9)
Age, week	22 ± 0.6	22 ± 0.5	21 ± 0.2
BW, g	23 ± 1.3	26 ± 1.5	29 ± 1.0*
HW, mg	105.4 ± 4	127.1 ± 11.4	144.7 ± 10.3*
HW/BW, %	0.46 ± 0.02	0.48 ± 0.03	0.49 ± 0.03
CF, ml min^−1^ g^−1^	16.7 ± 0.24	17.6 ± 1.07	15.9 ± 0.96
HR, beats min^−1^	356 ± 12	372 ± 8	384 ± 18
LVDP, mmHg	132 ± 17	106 ± 10	103 ± 9

Values are means ± SEM

*BW* body weight, *CF* coronary flow, *HFD* high-fat diet, *HR* heart rate, *LVDP* left ventricle developed pressure, *WT* wild type

*P < 0.05 vs. WT or ApoE KO

### Ex vivo effect of Up_4_A on CF and cardiac function in WT, ApoE, and ApoE + HFD mice

**Fig. 1:**
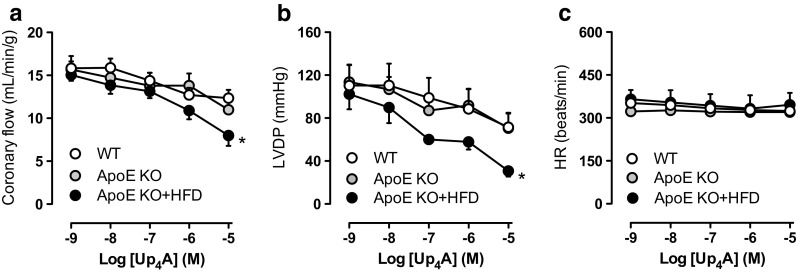
Effects of Up_4_A on coronary flow and cardiac function in atherosclerosis. Shown are Up_4_A concentration responses (10^−8^−10^−5^ M) in isolated hearts of wild-type (WT) (*n* = 6), ApoE knockout (KO) (*n* = 4), and ApoE KO + high-fat diet (HFD) mice (*n* = 5) for coronary flow (**a**), left ventricle developed pressure (LVDP, **b**), and heart rate (HR, **c**). Values are mean ± SEM. *Significant difference vs. concentration response curve in WT or ApoE KO by two-way ANOVA (*P* < 0.05)

Infusion of Up_4_A (10^−9^−10^−5^ M) into isolated hearts resulted in a concentration-dependent decrease in CF to a similar extent between WT (~ 34% maximal reduction from baseline at 10^−5^ M Up_4_A) and ApoE KO mice (~ 40% maximal reduction from baseline at 10^−5^ M Up_4_A). Notably, Up_4_A further and significantly decreased CF in perfused hearts of ApoE KO + HFD mice (~ 55% maximal reduction from baseline at 10^−5^ M Up_4_A, Fig. 1a). Likewise, Up_4_A infusion produced a concentration-dependent decrease in LVDP similarly between WT (~ 46% maximal reduction from baseline at 10^−5^ M Up_4_A) and ApoE KO mice (~ 48% maximal reduction from baseline at 10^−5^ M Up_4_A), which was further decreased in ApoE KO + HFD mice (~ 66% maximal reduction from baseline at 10^−5^ M Up_4_A, Fig. 1b). Infusion of Up_4_A (10^−9^–10^−5^ M) did not affect HR among the three groups (Fig. 1c).

### Effect of P2X_1_R antagonism on Up_4_A-mediated decreases in CF in ApoE and ApoE + HFD mice

**Fig. 2:**
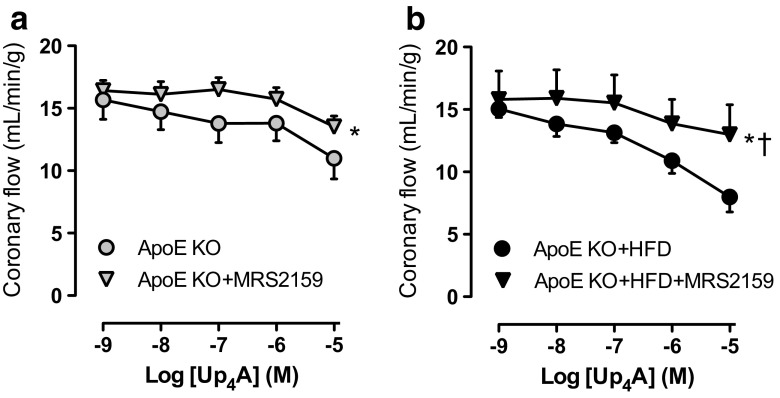
Effects of P2X_1_R inhibition on Up_4_A-induced decrease in coronary flow in atherosclerosis. Shown are the effects of P2X_1_R antagonist MRS2159 (30 μM) on Up_4_A concentration responses (10^−8^−10^−5^ M) in isolated hearts of ApoE knockout (KO) (**a**, *n* = 4 in ApoE KO; *n* = 5 in ApoE KO + MRS2159) and ApoE KO + high-fat diet (HFD) mice (**b**, *n* = 5 in ApoE KO + HFD; *n* = 4 in ApoE KO + HFD + MRS2159). Values are mean ± SEM. *Significant difference vs. corresponding control concentration response curve by two-way ANOVA (*P* < 0.05)

Considering that activation of P2X_1_R contributes to Up_4_A-induced vascular contraction in several vascular beds [5, 8] and the effects of Up_4_A on CF are comparable between WT and ApoE KO mice, the P2X_1_R antagonist MRS2159 was used to study potential involvement of the vasoconstrictor P2X_1_R in Up_4_A-mediated changes in CF from ApoE KO and ApoE KO + HFD groups. Up_4_A-induced decreases in CF in ApoE KO + HFD mice (ΔAUC: 19 ± 4, Fig. 2a) were significantly restored by MRS1259 to a greater extent than that in ApoE KO mice (ΔAUC 8 ± 2, Fig. 2b; *P* < 0.05). This indicates that the greater vasoconstrictor effect of Up_4_A observed in perfused hearts from ApoE KO + HFD mice is likely through greater activation of P2X_1_R.

### Immunohistochemistry for P2X_1_R expression in mouse coronary arteries in WT, ApoE, and ApoE + HFD mice

**Fig. 3:**
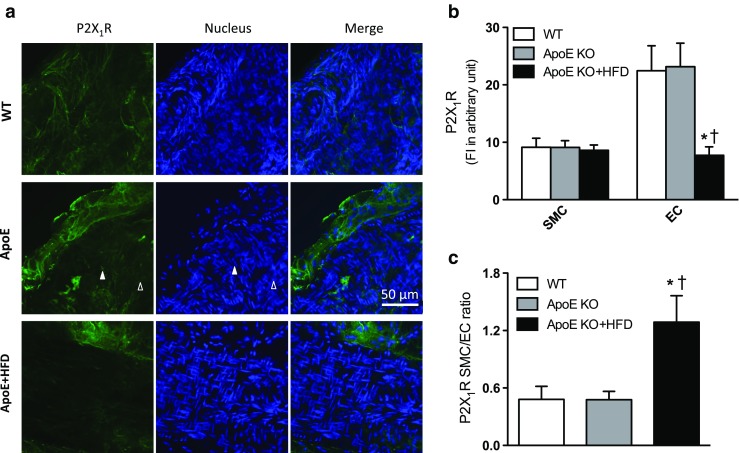
P2X_1_R expression in isolated mouse coronary arteries in atherosclerosis. **a** Representative confocal fluorescence images showing P2X_1_R fluorescence intensity in coronary arteries from WT, ApoE knockout (KO), and ApoE KO + high-fat diet (HFD) mice. *Scale bar* 50 μm; *solid triangle*: smooth muscle cells (SMCs) or corresponding nucleus; *open triangle*: endothelial cells (ECs) or corresponding nucleus. **b** Quantification of P2X_1_R expression in ECs vs. SMCs in isolated coronary arteries from WT (*n* = 5), ApoE KO (*n* = 9), and ApoE KO + HFD mice (*n* = 5). **c** SMC/EC ratio of P2X_1_R expression in isolated coronary arteries from WT, ApoE KO, and ApoE KO + HFD mice. Values are mean ± SEM. *P < 0.05 vs. corresponding WT; †*P* < 0.05 vs. corresponding ApoE KO

There is a generally accepted view that activation of purinergic receptors in ECs results in vascular relaxation, while activation of purinergic receptors in SMCs leads to vascular contraction [30]. We hypothesized that there is a greater upregulation of P2X_1_R in SMCs, thereby contributing to further decreases in CF in response to Up_4_A in ApoE KO + HFD mice. As shown in Fig. 3, coronary small arteries isolated from ApoE KO + HFD hearts exhibited a marked decrease in P2X_1_R expression in ECs as compared to either ApoE KO or WT mice, while there was no significant difference in P2X_1_R expression in SMCs among the three groups (Fig. 3a, b). Interestingly, the SMC/EC ratio of coronary P2X_1_R expression was greater in ApoE KO + HFD than ApoE KO or WT mice (Fig. 3c), suggesting a net vasoconstrictor potential of P2X_1_R in isolated coronary arteries of ApoE KO + HFD mice.

### In vivo effect of Up_4_A on CBF and cardiac function in WT, ApoE, and ApoE + HFD mice

**Fig. 4:**
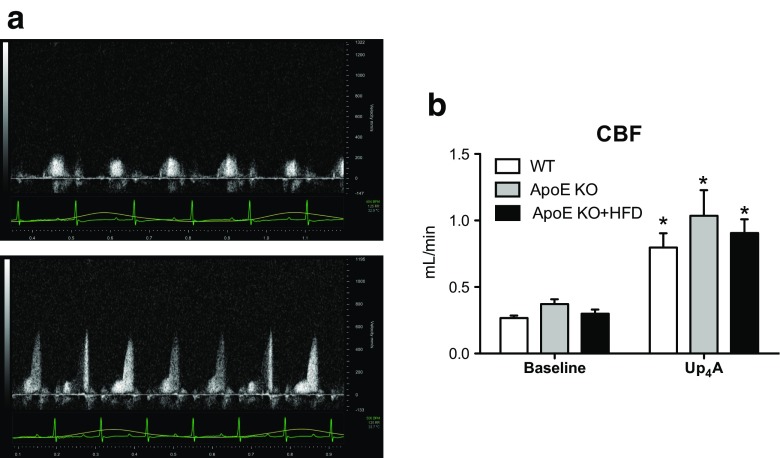
Effects of Up_4_A on coronary blood flow (CBF) in vivo in atherosclerosis. Shown are effects of bolus Up_4_A intravenous injection on CBF in WT (*n* = 5), ApoE knockout (KO, *n* = 3), and ApoE KO + high-fat diet (HFD) mice (*n* = 5). **a** Representative tracing for WT. **b** Quantification of baseline CBF and increase in CBF to a single dose of Up_4_A. Values are mean ± SEM. *P < 0.05 vs. corresponding baseline

**Fig. 5:**
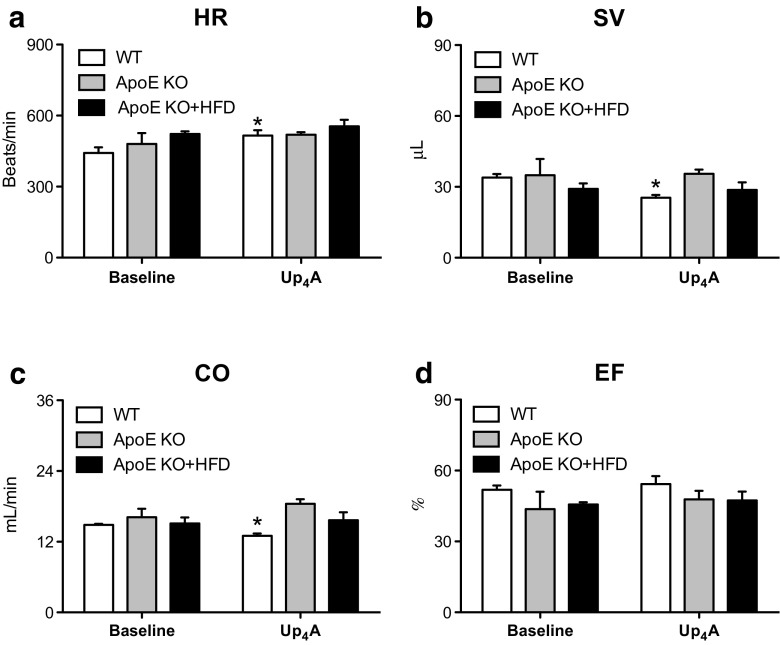
Effects of Up_4_A on cardiac function in vivo in atherosclerosis. Shown are effects of bolus Up_4_A intravenous injection on heart rate (HR, **a**), stroke volume (SV, **b**), cardiac output (CO, **c**), and ejection fraction (EF, **d**) in WT (*n* = 5), ApoE knockout (KO, *n* = 3), and ApoE KO + high-fat diet (HFD) mice (*n* = 5). Values are mean ± SEM. *P < 0.05 vs. corresponding baseline

To study the in vivo effect of Up_4_A on CBF, we used Doppler echocardiography to compare changes before and after Up_4_A bolus injection in anesthetized mice (Fig. 4a: an example of representative tracing for WT). Baseline CBF (expressed in mL/min) was not significantly different among the three groups (0.27 ± 0.02 in WT, 0.37 ± 0.04 in ApoE KO, and 0.30 ± 0.03 in ApoE KO + HFD; *P* > 0.05 by one-way ANOVA; Fig. 4b). Surprisingly, following an injection of Up_4_A (1.6 mg/kg, intravenously (i.v.)), CBF was significantly increased to a similar extent among the three groups (0.86 ± 0.11 in WT, 1.04 ± 0.19 in ApoE KO, and 0.91 ± 0.10 in ApoE KO + HFD; *P* > 0.05 by one-way ANOVA; Fig. 4b). There was no significant difference in baseline HR (Fig. 5a), stroke volume (SV, Fig. 5b), cardiac output (CO, Fig. 5c), and ejection fraction (EF, Fig. 5d) among the three groups. However, Up_4_A slightly but significantly increased HR (Fig. 5a), and, to a larger extent, decreased SV (Fig. 5b), which resulted in a significant reduction in CO in WT mice (Fig. 5c), while EF was not affected (Fig. 5d). There were no significant effects of Up_4_A on HR, SV, CO, and EF in either ApoE KO or ApoE KO + HFD mice as compared to corresponding baseline (Fig. 5a−d).

## Discussion

The main findings of the present study are: (1) Infusion of Up_4_A resulted in reduced CF in isolated hearts of WT and ApoE KO mice to a similar extent, while Up_4_A further decreased CF in ApoE KO + HFD mice; (2) P2X_1_R antagonism using MRS2159 restored impaired CF in ApoE KO + HFD more than that in ApoE KO mice; (3) The SMC/EC ratio of coronary P2X_1_R expression was greater in ApoE KO + HFD than ApoE KO or WT mice; and (4) In contrast, bolus injection of Up_4_A increased CBF in vivo to a similar extent among the three groups. The implications of these findings are discussed below.

Up_4_A was first identified as a potent endothelium-derived vasoconstrictor in rat perfused kidney [5]. Several subsequent studies have confirmed that Up_4_A produces vasoconstriction in rat renal arteries [31], aortas [10], gastric smooth muscle [32], and pulmonary arteries [11]. Furthermore, vasoconstriction was observed in mouse renal arterioles [12] and aortas [8]. On the other hand, there is evidence that Up_4_A can produce relaxation in isolated aortic rings of rats [10], rat kidney [15], and human and mouse colon [33]. Interestingly, the vascular effects or potency vary from mice to rats within the same type of arterial segments. Thus, Up_4_A produces a more pronounced contraction in rat aortas as compared to mouse aortas at basal tone [9, 10], whereas Up_4_A induces a concentration-dependent relaxation in rat aortas but produces a potent contraction in mouse aortas when the vessel tone is elevated [9, 10]. All these findings suggest that the vascular effects of Up_4_A may not only depend on different vascular beds but also different species studied [7]. In contrast to vasodilator effect of Up_4_A in porcine coronary small arteries (non-resistance vessel with diameter of ~ 250 μm) [6], infusion of Up_4_A into isolated mouse hearts (coronary resistance vessel) resulted in a concentration-dependent decrease in CF. Moreover, Up_4_A produces a mild contraction in isolated coronary arteries of mice (conduit arteries with diameter of ~ 100 μm; data not shown) further supporting the notion that Up_4_A-induced coronary vascular effect is species-dependent. Future studies are needed to explore the vascular effect of Up_4_A in human coronary arteries with a similar diameter setting.

We and others have previously demonstrated altered purinergic signaling in coronary microcirculation in atherosclerotic/-like animal models. Thus, ATP increases CBF, which is reduced in ApoE KO mice, likely via decreased activation of P2Y_2_R [34]. A_2A_R agonist increases CF in both ApoE KO and ApoE KO + HFD mice through increased activation of A_2A_R [19], while coronary reactive hyperemia is impaired in female ApoE KO + HFD as compared to WT mice, suggesting a compensatory mechanism for adenosine receptor-mediated coronary microvascular regulation in atherosclerosis [20]. Moreover, although coronary vasodilation is not altered in response to adenosine receptor stimulation, A_2B_R is downregulated in coronary arterioles of swine with early-stagy metabolic syndrome [35]. P1Rs are the major purinergic receptors involved in Up_4_A-mediated coronary relaxation in swine. Up_4_A-induced coronary relaxation is blunted in swine following myocardial infarction, likely through downregulation of A_2B_R [17]. In the present study, infusion of Up_4_A into isolated mouse hearts resulted in a comparable reduction in CF between ApoE KO and WT mice, while Up_4_A further decreased CF in ApoE KO + HFD mice (Fig. 1a). The further decrease in CF in ApoE KO + HDF mice was possibly through increased activation of P2X_1_R, as evidenced by the rescue of P2X_1_R antagonism on CF is higher in ApoE KO + HFD as compared to ApoE KO mice (Fig. 2). Additionally, the comparable reduction in CF response to Up_4_A between WT and ApoE KO mice and further decrease in CF in ApoE KO + HFD are supported by a similar P2X_1_R expression pattern in the coronary arteries of mice (Fig. 3); particularly, P2X_1_R is proposed to be more abundantly expressed in arterioles [36]. Thus, no differences in coronary P2X_1_R expression in ECs between ApoE KO and WT mice were noted; P2X_1_R expression in ECs was markedly decreased in ApoE KO + HFD mice. The P2X_1_R expression in SMCs of mouse coronary arteries is similar among the three groups. It is generally accepted that activation of P2 receptor subtypes in ECs leads to vasodilation while activation of P2 receptor subtypes in SMCs results in vasoconstriction [30]. Interestingly, the SMC/EC ratio of coronary P2X_1_R expression was greater in ApoE KO + HFD than ApoE KO or WT mice (Fig. 3c), suggesting a greater net vasoconstrictor potential of P2X_1_R in isolated coronary arteries of ApoE KO + HFD mice, contributing to a further reduction in CF in response to Up_4_A. The alteration of P2X_1_R expression in ECs but not SMCs is likely due to influence of atherosclerotic lesions in coronary vasculature in our ApoE KO + HFD mice [37]. Future studies are needed to confirm this issue. Interestingly, we previously demonstrated that an endothelial dysfunction (evaluated by A23187 concentration response-induced nitric oxide-dependent flow increase and reactive hyperemia) exists in coronary microcirculation in female but not in male ApoE KO + HFD mice, while sodium nitroprusside-induced increase in coronary flow is comparable between WT and ApoE KO + HFD groups in both sexes [20]. In isolated hearts from male mice of the present study, Up_4_A still further decreased coronary flow in ApoE KO + HFD compared to WT mice (Emax 13.4 ± 0.8 mL/min/g in WT vs. 6.0 ± 0.8 mL/min/g in ApoE KO + HFD; *P* < 0.05 by two-way ANOVA, *n* = 3), suggesting Up_4_A-induced decrease in coronary flow is likely direct rather than via an indirect effect of endothelial dysfunction.

Together with the functional role of P2X_1_R in Up_4_A-mediated vasoconstriction in perfused rat/mouse kidney [5, 12] and isolated mouse aortas [8], this evidence suggests that there is likely a similar activation of P2X_1_R in Up_4_A-induced decreases in CF in WT mice as compared to ApoE KO mice. We previously showed that more excessive atherosclerotic lesions and greater total cholesterol levels were present in ApoE KO + HFD mice, followed by ApoE KO mice when compared to WT mice [19–21]. Moreover, coronary atherosclerotic lesions were formed in left coronary arteries of ApoE KO + HFD mice (unpublished). However, CF in response to Up_4_A and coronary P2X_1_R expression are apparently not affected by more excessive atherosclerotic lesions and greater total cholesterol conditions in ApoE KO mice as compared to WT mice (Figs. 1a and 3). The further reduction in CF induced by Up_4_A, as well as upregulation of the net vasoconstrictor P2X_1_R in ApoE KO + HFD mice, may be due to advanced atherosclerosis. In line with this concept, several P2XRs including P2X_1_Rs are upregulated in bladder tissue that underwent atherosclerosis-induced ischemia [38]. This implies a crucial role for P2X_1_R in an advanced atherosclerotic model.

The in vivo effect of Up_4_A has been shown by Hansen et al., where i.v. infusion of Up_4_A (512 nmol/kg/min) into conscious mice [14] induces hypotension, suggesting a vasodilator influence of Up_4_A on systemic circulation. The Up_4_A-induced systemic vasodilation is direct, rather than indirect, through degraded product (e.g., adenosine) and contradictory to vasoconstrictor effects observed in most isolated arteries from systemic vascular beds [7]. In the present study, bolus injection of Up_4_A resulted in more than a twofold increase in CBF as compared to baseline in WT mice (Fig. 4a, b), which was similar to ApoE KO or ApoE KO + HFD groups. The dose of Up_4_A (1.6 mg/kg ≈ 40 μg/animal) used in the present study was much higher than that used in the study by Hansen et al. (512 nmol/kg/min ≈ 0.3 μg/animal) [14]; thus, i.v. injection of such high dose Up_4_A possibly induces systemic vasodilation and hypotension. This may result in an increase in HR and decreases in SV and CO in WT mice observed in the present study.

The different effects of Up_4_A on CF between ex vivo and in vivo cannot be readily explained. The in vivo system is much more complex compared to isolated hearts in which there is no influence of hormone, nerves, blood flow, and blood components. The increase in HR in response to Up_4_A in WT mice may increase metabolic demand, leading to increases in CBF. However, the changes in cardiac function induced by Up_4_A observed in WT mice are not present in ApoE KO or ApoE KO + HFD mice (Fig. 5), while the increase in CBF is comparable among the three groups (Fig. 5b), suggesting that the changes in metabolic demand unlikely account for the increased CBF. Another explanation is that the vasodilator effect of Up_4_A on coronary circulation is indirectly through nucleotidase-breakdown products, e.g., ATP, as activity of nucleotidase such as ATPase and ADPase in aortas of ApoE KO mice is altered [34]. However, Hansen et al. proposed that degradation of Up_4_A is unlikely by continual infusion into systemic circulation [14]. The net vasodilator effect of Up_4_A observed in vivo may be due to the indirect vasodilator effects from Up_4_A-activated vasodilator purinergic receptors in non-vessel tissues (e.g., erythrocyte) overweighing the direct vasoconstrictor effects from Up_4_A-activated vasoconstrictor purinergic receptors in coronary vasculature. As mentioned earlier that the dose of Up_4_A used in the present study is high, the Up_4_A-induced increase in CBF may, however, still not plateaued as compared to a greater coronary vasodilation induced by adenosine in mice from previous studies of ours [28] and others [39]. This may explain why the increases in CBF in response to Up_4_A among WT, ApoE KO, and ApoE KO + HFD groups are not significantly different.

In conclusion, the present findings demonstrate that there are divergent vascular effects of Up_4_A on coronary microcirculation between ex vivo and in vivo in mice. Up_4_A directly decreases CF, possibly through P2X_1_R, which is further reduced via greater downregulation of the vasodilator P2X_1_R in ECs in ApoE KO + HFD mice. In contrast, Up_4_A increases CBF in vivo regardless of the atherosclerotic model. Based on the dysregulation of P2X_1_R in coronary microvasculature in atherosclerosis, P2X_1_R may serve as a potential therapeutic target for the treatment of ischemic heart disease.
